# Preventing suicide: a resource for the family

**DOI:** 10.1186/1744-859X-7-1

**Published:** 2008-01-24

**Authors:** Sergio A Pérez Barrero

**Affiliations:** 1 Medical University of Granma, Cuba

## Abstract

The family can play an important role in the prevention of suicide if it is capable of aiding the mental health care services in the early detection and management of family members at risk. In order to attain this goal, the whole family should be informed in how to prevent suicide.

## Background

Suicide is one of the 10 major causes of death in most countries. The family can play an important role in its prevention, as it is an avoidable cause of death. In order to be able to prevent suicide among its members, the family should rid itself of some myths associated with suicidal behavior.

Myths can be defined as culturally accepted phenomena rooted in the minds of people that do not reflect any scientific truth; in the case of suicide they are erroneous judgments concerning the act itself and the person who takes their own life. Such myths need to be removed if people at risk are to be helped.

Myths tend to justify their advocates' attitudes and become a hindrance in the prevention of suicide. There are many myths in relation to suicide and the suicides. We will consider some of these, and also explain some scientific criteria that should be taken into consideration by the family in order to help prevent suicide among its members.

### Myth 1: those who want to end their life will not admit it

This myth leads to people not paying attention to those who do express their suicidal ideas or threaten to commit suicide.

A total of 9 out of 10 people who committed suicide expressed their purposes clearly, and it is likely 10 in 10 people who commit suicide will have hinted at their intention to put an end to their live.

### Myth 2: those who says they will do it, will not

This myth leads to suicide threats not taken seriously because they are taken as blackmail, manipulation, bluff, etc.

In fact, every person who commits suicide announces with words, threats, gestures or changes of behavior what is about to happen.

### Myth 3: a person who will commit suicide does not give any hints about what he or she is up to

This myth tries to ignore the prodromic manifestations of suicide. However, as stated for myth 2, every person who commits suicide announces with words, threats, gestures or changes of behavior what is about to happen.

### Myth 4: those who attempt suicide are cowards

This myth tries to avoid consideration of the true causes of suicide by attributing the behavior to a negative personality trait.

In fact, those who commit suicide are not cowards, but people who are suffering.

### Myth 5: those who attempt suicide are courageous people

This myth tries to attribute suicidal behavior to a positive personality trait. This criterion hinders suicide prevention because it portrays suicidal behavior as justified, as it is considered synonymous to bravery, an asset that everybody would like to possess.

However, those who attempt to commit suicide are neither brave people nor cowards, as bravery and cowardice are personality traits that cannot be quantified or measured by the number of times you attempt to kill yourself or decide to give yourself another chance.

### Myth 6: asking a person at risk if they have thought of committing suicide could stimulate them to do it

This myth instills a fear of speaking about the topic of suicide with people who are at risk of committing it.

It has been proven that talking about suicide with a person at risk does not stimulate the idea itself, but instead contributes to reducing the likelihood of the act and it might be the only possibility offered to the subject for analysis of his or her self-destructive reasoning [1,2].

## Suicide risk groups

In addition to the myths about suicide, the family should also learn about suicide risk groups.

Suicide risk groups are groups of people, who according to their particular characteristics, could be at greater risk of committing suicide than the general populace. Major suicide risk groups include the depressed, subjects who have made previous suicide attempts, subjects who have suicidal ideas or have threatened to commit suicide, survivors (see below for definition), and vulnerable subjects facing a crisis. Below, we will briefly describe each group.

### The depressed

Depression is a common disease related to people's moods. The most common symptoms are sadness, lack of motivation to do things, lack of will, desire to die, multiple somatic complaints, suicidal ideation, suicidal acts, sleep and appetite disorders, and carelessness about personal hygiene.

Some characteristics of adolescents' depressive state of mind are as follows.

• They tend to be more irritable than sad.

• Fluctuations of their affective behavior are more frequent than in adults, whose moods tend to be more stable.

• Hypersomnia is more frequent than insomnia.

• They are more likely to complain of physical symptoms when they feel depressed.

• They are more prone to exhibit episodes of violence and antisocial behavior as a manifestation of mood disorders than adults.

• They might show risky behavior, e.g. alcohol or drug abuse, or driving motor vehicles at high speeds while either sober or drunk.

• The likelihood of committing suicide is higher in adolescents than in adults in similar situations.

In the elderly, depression can appear disguised as:

#### Depression as normal aging

In this case, the older person loses interest in the things they used to like most, lack vitality and willpower, tend to relive the past, lose weight, suffers from sleep disorders, complains about memory impairment, and will have a tendency to live in isolation (e.g. they will spend most of their time in their bedroom). For many people this picture is a normal behavior for old people and not an indication of depression.

#### Depression as abnormal aging

In the elderly, different degrees of disorientation to times, places and people might be present; they might confuse people they know with each other, they are not able to recognize places, there is a deterioration of their abilities and control over habits, sphincter relaxation appears (i.e. the old person urinates and/or defecates uncontrollably), they might present gait impairments that resemble cerebrovascular disease and so on. They might also suffer from behavior disorders, for instance, refusing to be fed, etc. For many people, this picture is consistent with irreversible dementia and not an indication of depression.

#### Depression as physical, somatic or organic disease

Old people complain of multiple physical symptoms, such as headache, backache, chest pain or pain in the legs. They might also complain of digestive disturbances such as slow digestion, heartburn, or abdominal bloating even without having eaten anything. They take laxatives, antacids and other medications to get relief for their gastrointestinal disturbances, they complain of losing their taste sensation, they lack appetite, they lose weight, they have cardiovascular problems such as palpitations, oppression, breathlessness, etc. For many people this picture is consistent with a somatic disease and not an indication of depression.

#### Depression as a non-depressive mental disease

Old people often have the feeling that someone is watching or following them, that someone wants to kill them, or that everybody is talking about them. When they are asked why they think this, they answer that they deserve it because "they are the worst human beings on earth", "the greatest of all sinners", and similar expressions that indicate depression.

#### Depression as a depressive mental disease

This is characterized by the following.

• A depressive state of mind most of the day and/or every day.

• Marked reduction of pleasure or interest in all or most of their daily activities.

• Loss of weight without going on a diet, or weight gain of about 5%.

• Daily insomnia or hypersomnia.

• Psychomotor agitation or retardation.

• Daily fatigue or lack of energy.

• Inappropriate feelings of guilt, which can lead to guilt delusion.

• Decreased capacity to think or to concentrate and hesitancy during most of the day.

• Recurrent thoughts of death or suicide.

As we can see, it is not wise to infer that any symptom presented by old people is simply due to their age and the ailments that characterize that period of life, to dementia, or to a physical illness. Such symptoms can be manifestations of depression and, consequently, vitality and the remaining compromised functions can be recovered if the depression is treated. If depression is not properly diagnosed, it can become chronic and it can lead to suicide [3,4].

### Subjects who have made previous suicide attempts

According to some studies, 1–2% of those who had made a suicide attempt committed suicide during the first year that followed the attempt, and 10–20% committed suicide at a later point in their lives.

### Subjects who have had suicidal ideas or have threatened to commit suicide

Having suicidal ideas does not necessarily lead to committing suicide. Several studies have reported individuals who had had suicidal ideas during their lives and never experienced an act of self-aggression. However, when suicidal ideas appear as a symptom of mental disorder and they are accompanied by a high suicidal tendency, an increasing frequency, and a detailed planning in circumstances that favor the act, the risk of suicide is very high.

### Survivors

Survivors in this sense are those people who have very close links with a person who dies as a result of suicide. Among the survivors are relatives, friends, partners, and even the doctor, psychiatrist, or any other therapist who attended to the deceased.

### Vulnerable subjects facing a crisis

This group includes mainly non-depressed mental patients such as schizophrenic and/or alcoholic patients, drug addicts, anxious people, people with personality disorders, and those with impulse control disorders. This group also includes individuals who suffer from a terminal, malignant, painful or disabling physical illness that jeopardizes their quality of life.

This group also includes certain groups of individuals, such as ethnic minorities and immigrants, who are not able to adapt themselves to their new country of residence, who are considered second-class citizens, and those who have been tortured or have been victims of violence of some form [5,6].

When such individuals face a conflict or a significant event beyond their capacity to solve, they tend to resort to suicide. When subjects from any risk group are in crisis, they can communicate their suicide intentions in different ways. For instance, the subject might threaten to commit suicide, or say that:

• he/she wishes to kill himself/herself;

• he/she wants to die;

• other people would feel better if he/she did not exist;

• it is preferable to be dead than alive;

• he/she has had bad ideas;

• the rest of the world will not have to stand him/her any longer;

• he/she does not want to live;

• it is preferable to be dead than to live his/her life;

• he/she has thought about putting an end to his/her life;

• his/her life should not be lived;

• he/she does not want to be a burden on other people;

• his/her life is not worth living;

• he/she would like to fall asleep and never wake up;

• he/she is tired of living [7,8].

As the family becomes aware of the many different forms that risk of suicide can be communicated, they should also learn to identify the situations that can lead to suicide risk in order to increase family support. Among these situations are the following.

### In childhood

• Watching painful events (domestic violence);

• familial break-up;

• the death of a loved one who provided emotional support;

• living with a mentally ill person as the only next of kin;

• having been scolded in a humiliating way.

### In adolescence

• Facing a troubled love life;

• having a damaged relationship with significant figures (father, mother, or teacher);

• parents' expectations and demands beyond the reach of adolescents' capacities;

• unwanted pregnancies;

• concealed pregnancies;

• examination periods;

• having friends who exhibit suicidal behavior or consider suicide as a way to solve problems;

• love disappointments;

• the "hustle and bustle" of modern life phenomenon;

• having been scolded in a humiliating way;

• sexual abuse or harassment perpetrated by significant figures;

• loss of significant figures as a result of marriage break-up, death or abandonment;

• periods of adaptation to military regimens or boarding school systems;

• awareness of serious mental disease.

### In adulthood

• Unemployment (during the first year of job loss);

• having a competitive wife (in some male-oriented cultures);

• public personalities involved in sexual scandals (politicians, religious people, etc.);

• bankruptcy;

• recent psychiatrist hospitalization;

• hospital discharge with a serious mental disease.

### In old age

• Initial period of institutionalization;

• first year after death of lifelong partner in men and second year in women;

• physical and psychological abuse;

• physical illnesses that affect sleeping (chronic insomnia);

• loss of mental capacity [9-11].

## Dealing with the potentially suicidal

In the presence of a subject belonging to one of the risk groups mentioned, who is facing any of the situations described it is essential to carry out a thorough exploration of their suicidal ideation. The following are variants to approach this topic.

### First variant

You can ask the family member at risk, "Obviously you are not feeling well. I have noticed that, and I would like to know how you think you might solve the problem".

In this option, an open question can be asked to give the subject the opportunity to express his/her thoughts so that his/her suicidal tendencies can be exposed.

### Second variant

Questions can be asked based on the symptom or symptoms that most annoy the subject to discover any suicidal tendencies. For instance, "You say you have difficulty sleeping, and I know that when it happens sometimes strange ideas cross your mind. Would you like to talk about them? What do you think about when you cannot sleep?".

### Third variant

The subject can also be approached in this way: "I know you have not been feeling well lately. Have you had any bad thoughts?".

In this case, "bad thoughts" is synonymous with suicidal ideas. It is also possible to use expressions such as "unpleasant thoughts", "recurrent" or "strange" thoughts, etc. If the subject answers affirmatively, the questioner should try to find out what those bad thoughts are, as they might be associated with unjustified fears such as the fear of becoming diseased or receiving bad news, which are not necessarily suicidal ideas.

### Fourth variant

The subject can be asked directly whether he or she has had suicidal ideas, such as "Have you considered killing yourself as a solution to all your problems?", "Have you thought about committing suicide?", "Has the idea of ending your life ever crossed your mind?".

### Fifth variant

It is necessary that both the subject at risk and the questioner know of a previous case of suicide committed by a family member, friend or neighbor. The question should be asked in this way "Are you thinking of solving your problems by ending your life like xxxx did?".

If the answer to this question suggests that the subject has suicidal ideas, it is advisable to continue asking the following sequence of questions.

### How do you plan to do it?

This question is intended to find out the suicide method being considered. Any method can be lethal. Suicide risk is greater if there are previous cases of suicide committed by other family members using the same method. The risk is even greater in cases of repeat suicide attempts, where the suicidal person might be in search of a more lethal (i.e. successful) suicide method. For the prevention of suicide it is vital to avoid the availability of or access to methods that could inflict harm to the subject.

### When do you plan to do it?

This question does not aim to get an exact date of when the person plans to commit suicide, but is intended to find out if the subject is making arrangements, for example, to bequeath their possessions or whether he/she has written farewell notes, if he/she is giving away valuable items, if the person expects a significant event to take place such as the break-up of an important relationship, the death of a beloved person, etc.

Subjects at risk of committing suicide should always be in the company of someone else, as being alone increases the likelihood the act will be accomplished.

### Where do you plan to do it?

This question might lead to discovery of where the subject has thought they might commit suicide. The act usually takes place in a spot visited by the suicidal person on a regular basis, mainly his or her home, school, or the home of a family member or friend. Other high-risk locations are distant places (e.g. countryside), places hard to find or places that have been used before in other suicides.

### Why do you want to do it?

This question tries to find out the motive or reason for why the subject wants to commit suicide. Among the most common motives are troubled relationships, academic problems, having been scolded in a humiliating way, etc. Motives should always be considered significant for the subject at risk and they should never be dismissed or appraised from the point of view of other family members.

### What do you want to do it for?

The aim of this question is to find out the meaning of the suicidal act to the person. Wishing to die is the most dangerous motive, but not the only one. There could be other reasons involved, such as attracting other people's attention, to show the magnitude of their problems, to express rage or frustration, to ask for help, to attack others, and so on [2,12].

## Conclusion

The more questions the subject can answer, the better shaped his suicidal plan is and the higher the risk. Consequently, the following question is raised: what should the family do when one of its members has suicidal ideas?

There are four main measures to undertake:

• Never leave him/her alone.

• Ensure the method chosen by the subject cannot be used.

• Make all family members aware of the subject's suicide crisis so that they can help to keep an eye on the subject and to provide emotional support.

• Contact a mental health institution so that the subject can receive specialized professional care.

It should be remembered that a suicide crisis can last hours, days, and in rare cases weeks, so the main goal is to keep the subject alive until he/she can receive specialized care. Never forget that suicide is a death that can be avoided.
